# Cytotoxic and Radiosensitizing Effects of Folic Acid-Conjugated Gold Nanoparticles and Doxorubicin on Colorectal Cancer Cells

**DOI:** 10.34172/apb.2022.079

**Published:** 2021-10-06

**Authors:** Behnam Heshmatian, Zhaleh Behrouzkia, Mahshid Mohammadian, Zhino Moradi, Zeinab Mohammadi, Reza Zohdi Aghdam

**Affiliations:** ^1^Department of Physiology, Faculty of Medicine, Urmia University of Medical Sciences, Urmia, Iran.; ^2^Medical Physics Department, Faculty of Medicine, Urmia University of Medical Sciences, Urmia, Iran.; ^3^Department of Biochemistry, School of Medicine, Urmia University of Medical Science, Urmia, Iran.

**Keywords:** Doxorubicin, Gold nanoparticle, X-Ray irradiation, HT-29 cells

## Abstract

***Purpose:*** Radiotherapy is one of the most important therapeutic options used to treat cancers. Radiation effects can be improved using nanoparticles and chemotherapeutic drugs as radiosensitizing agents. The aim of the present study was to evaluate the effects of folic acid-conjugated gold nanoparticles (GNP-F) in combination with doxorubicin (DOX) and x-Ray irradiation in colorectal cancer (CRC) cell line (HT-29).

***Methods:*** The cell viability assay (WST-1) was performed to study the cytotoxic effects of different concentrations of DOX and GNP-F after 24 and 48 hours treatments. Then, the effects of the GNP-F, X-Ray irradiation, and DOX drug in single and combined treatments were examined after 24 and 48 hours treatment with effective doses. Likewise, the caspase 3 gene expression ratio and the caspase 3 activity were assessed after 48 h treatment. Moreover, the *malondialdehyde (MDA) level* was determined in treated and untreated cells.

***Results:*** When GNP-F (at a concentration of 70 μM) was combined with X-ray irradiation (2 Gy) and DOX drug, induced more cytotoxic effects compared to the control group. The results of cell viability assay showed that GNP-F + X-Ray in combination with a low concentration of DOX (0.25 × IC50) enhanced the cytotoxic effects of cells compared to related single treatments. Caspase 3 gene expression ratio and caspase 3 activity increased in double and triple combination treatments in comparison with the single groups. Moreover, the MDA level increased in triple combination compared to the single treatments.

***Conclusion:*** Our findings confirmed the potential anti-cancer effects of the GNP-F and DOX in combination with X-Ray irradiation in CRC cells.

## Introduction

Colorectal cancer (CRC) is one of the most common cancers affecting people worldwide.^1,2^ Among different cancer therapeutic strategies, radiation has an important role in cancer treatment, but radiation resistance remains a cause of failure in achieving successful results.^3,4^ One of the therapeutic strategies to enhance the radiation effects in cancer cells and overcome their radiation resistance is the use of a radiosensitizer agent.^3^

Resistance to chemotherapy drugs is also another main problem in the effective treatment of several cancers.^5^ Doxorubicin (DOX), an anthracycline antibiotic is a chemotherapeutic agent, which is most widely utilized in the treatment of cancer alone or in combination with other anti-cancer drugs or radiation therapy.^6^ At the cellular level, DOX displays various molecular mechanisms that are related to interference with DNA unwinding, inhibition of the respiratory chain enzymes in mitochondria, and apoptosis induction in response to inhibition of topoisomerase II.^7^ Nevertheless, a reduction in the effects of chemotherapeutic drugs such as DOX is characterized as drug resistance. Multiple molecular and cellular mechanisms are related to drug resistance. In this regard, drug resistance, predominantly multidrug resistance, is considered as one of the main obstacles to effective chemotherapy.^8,9^

The therapeutic outcome in CRC tumors is very low due to drug resistance to DOX.^10,11^

Nowadays nanoparticles, could be applied for cancer therapy, and increase intracellular uptake of DOX with decreased side effects versus conventional DOX.^12^

The potential value of nanoparticles as new radiosensitizers has been revealed.^3^

Gold nanoparticles (GNPs) have been applied effectively as radiation sensitizing agents. Previous studies demonstrated that GNPs can sensitize cancer cells, including cancer cell lines, to radiation.^13-15^ Besides, it has been indicated that the GNP and other metal nanoparticles can be used as drug carriers for DOX and other agents.^16-19^ Definitely, HT29 cells as CRC cells, exhibit cellular resistance to radiotherapy.^20^ chemoradiation resistance has been related with the levels of the apoptosis inhibitors in various cancers.^21^ Accordingly,the purpose of the present study was to evaluate the radiation-sensitivity effect of folic acid conjugated-gold nanoparticles (GNP-F) in combination with DOX chemotherapeutic agent in HT29 cell line and assessment of possible underlying mechanisms of these therapeutic agents in CRC cells.

## Materials and Methods

###  Materials 

 Dulbecco’s modified eagle’s medium (DMEM), fetal bovine serum (FBS), penicillin, and streptomycin were purchased from Biowest (France). Water Soluble Tetrazolium Salts (WST-1) kit was prepared from TAKARA (Japan). DOX hydrochloride and phosphate-buffered saline were obtained from Sigma-Aldrich. dimethylsulfoxide was purchased from ATOCEL (Austria). A stock solution of DOX (12.5 mg/mL) was prepared in water and stored at 4°C.

###  Cell culture and treatments 

 HT-29 CRC cells were obtained from the Iranian Biological Resource Center (Tehran, Iran) and harvested in DMEM, supplemented with 10% FBS and 1% penicillin/streptomycin (Biowest, France). The study protocol was reviewed and approved by the ethical committee of Urmia University of Medical Sciences.

GNP-F was purchased from the R&D department of Nanobon Company (Tehran, Iran). Based on the Nanobon company protocol for preparing GNP-F, an aqueous solution of *trisodium citrate* and HAuCl4 (Au; ppm) were heated and then stirred until the solution color was turned to deep red. In the next step, the solution of folic acid (in ethanol) was added and sonicated. The GNP-F had *spherical* morphology and the average particle size was 40 nm.

GNP-F and DOX were diluted in DMEM and then mixed. Subsequently, for the assessment of drug response, 1 × 10^4^cells were cultured in a 96-well plate (24 hours before the treatment). The HT-29 cell line was treated with different concentrations of DOX (40, 20, 10, 5, and 2.5 µM) for 24 and 48 hours. The IC_50_ value was measured by CompuSyn software. The IC_50_ values of DOX after 24 and 48 hours treatment were equal to 10.8 and 8.6 µM, respectively.

To evaluate the cytotoxic effects of GNP-F in HT-29 cells, different concentrations of GNP (200, 100, 70, and 35 µM) were prepared and exposed to the HT-29 cells for 24 and 48 hours. According to the dose-response curves, the GNP-F at a concentration of 70 µM induced minimal toxic effect compared to other examined concentrations. So, this concentrationof GNP-F** (**70 µM) was selected for all treatments in single, double, and triple combined treatments.

For radiation treatment, 1 × 10^4^ HT-29 cells were irradiated with a clinical linear accelerator machine (Elekta Compact 6 MV, radiotherapy unit, Emam Khomeini hospital, Urmia, Iran) to single-dose irradiation (2 Gy) at a dose rate of 200 cGy/min with a field size of 10 cm × 15 cm and source-to-surface distance (SSD) = 100 cm at room temperature.

The double-combination treatments with DOX (DOX + GNP-F and X-ray + DOX) were tested at the concentration of 0.5 × IC_50_ (concentrations of DOX were 5.4 and 4.3 µM for 24 and 48 hours treatment times, respectively). The cytotoxic effects of triple combination (DOX + X-Ray + GNP-F) also were examined at a concentration of 0.25 × IC_50_ DOX (concentrations of DOX were equal to 2.7 and 2.15 µM for 24 and 48 hours treatment, respectively). GNP-F at the concentration of 70 µM and 2 Gy of X-Ray irradiation were utilized in all treatments. Each test was performed in triplicate.

 The cell viability assay kits were obtained from TAKARA BIO INC (Japan). The water-soluble tetrazolium-1 (WST-1) assay is based on the cleavage of the tetrazolium salt to dark red formazan. Cell viability assay was performed after 24 and 48 hours in treated and untreated cells. In this way, 10 μL of WST-1 solution was added to each well. After two hours of incubation, the absorption rate was determined at 450 nm with a reference wavelength level of 650 nm using the enzyme-linked immunosorbent assay microplate reader. In further experimental analysis, including caspase 3 gene expression, caspase 3 protein activity, and MDA level assays, DOX at the concentrations of IC50 (8.6 µM), 0.5 × IC50 (4.3 µM), and 0.25 × IC50 (2.15 µM) were used in single, double and triple combination treatments, respectively (after 48 h of treatment). Also, in all treatments, 2 Gy of X-ray irradiation and a concentration of 70 µM GNP-F were used.

###  Real-time polymerase chain reaction (PCR)

1 × 10^6^ of HT-29 cells were cultured in the absence or presence of DOX, GNP-F, and radiation at 0.5 × IC_50_ and 0.25 × IC_50_ concentrations of DOX in double and triple combination treatments, respectively. After 48 hours treatment, total cellular RNA was extracted based on the protocol described with the Gene All Kit (South Korea). RNA integrity was assessed by electrophoresis in a 1.0% agarose gel. Total RNA was reverse transcripted into cDNA. Indeed, cDNA synthesis was performed by the cDNA synthesis kit (GeneAll, South Korea). Real-time PCR was carried out using Real Q Plus 2x Master Mix Green (Ampliqon) according to the kit protocol using specific primers of β-Actin and caspase 3. Real-time PCR was completed at 35 cycles of denaturation for 30 seconds at 95°C, annealing for 30 seconds (59°C for both genes), and extension for 30 seconds at 72°C. The threshold cycle (Ct) values were assessed and the relative expression levels of mRNA were measured via the 2^-ΔΔCt^ method.

###  Caspase 3 activity

The activity of caspase 3 was determined using the caspase 3 colorimetric assay kit according to the manufacturer’s recommended protocol (Abnova). Briefly, after 48 h, untreated and treated cells [DOX at IC50 (8.6 µM), 0.5 × IC_50_ (4.3 µM), and 0.25 × IC_50_ (2.15 µM) concentrations were utilized in single, double, and triple combination treatments, respectively] were collected and kept on ice. Then, 50 μL of chilled cell lysis buffer was added to each treatment, incubated for 10 minutes on ice, and centrifuged. In the following, the supernatant was transferred to the fresh tube and kept on ice. The protein concentrations of the supernatants were measured by the Bradford technique. Then, 100 μg protein was diluted to 50 μL cell lysis buffer for each assay. In the following, 50 μL of 2X reaction buffer and 5 μL of DEVD-*p*NA substrate were added to each assay. The samples were incubated at 37°C for 2 hours and read at the wavelength of 405 nm. The changes in caspase 3 activity were studied by comparing these results with the level of uninduced control.

###  Assessment of malondialdehyde (MDA) levels 

To detect the lipid peroxidation rate, as indicated by MDA level, supernatants of untreated and treated cells [after 48 hours of exposures] in single, double, and triple combination treatments as described above, were collected and kept on ice and measured by the thiobarbituric acid (TBA) reaction based on the previously described procedure.^22^ Briefly, 3 mL orthophosphoric acid (1% v/v)

 was added to the supernatant and mixed (by Vortexing). In the following, 2 mL of 6.7 g L−1 TBA was added and heated at 100°C (45 minutes) then chilled. In the next step, N-butanol was added and centrifuged. The absorbance rate was measured spectrophotometrically at the wavelength of 532 nm. MDA concentrations were determined based on the simultaneously prepared calibration curves by MDA standards.

###  Statistical analysis

All tests were carried out in three replicate experiments. Data were shown as the mean ± SD. Statistical analysis was performed by the one-way ANOVA by GraphPad Prism version 4.0 (GraphPad Software Inc., San Diego, California). *P <* 0.05 was considered as the significance level. For the evaluation of IC_50 _values, the Compusyn software was used.

## Results and Discussion

**Figure 1:**
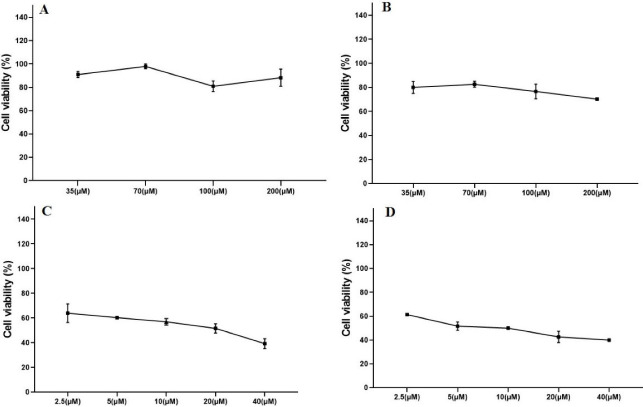
The cytotoxic effects of various concentrations of GNP and DOX using cell viability assay (WST-1). GNP-F at the concentrations of 200, 100, 70, and 35 µM after 24 h (**A**) and 48 h treatments (**B**) and DOX at 40,20,10,5, and 2.5 µM concentrations after 24 h (**C**) and 48 h treatments (**D**) in HT-29 cells. Data were presented as mean ± SD. GNP; Folic acid conjugated-gold nanoparticles, DOX; Doxorubicin

In order to evaluate the cytotoxicity of various concentrations of single DOX and GNP-F, the cell viability assay was carried out and dose-response plots for both agents were drawn. According to our results, the exposure of HT-29 cells to the different concentrations of single DOX decreased the cell viability after 24 and 48 hours treatments (Most of the studied concentrations). The results of the WST-1 analysis were plotted in Figure 1.

**Figure 2:**
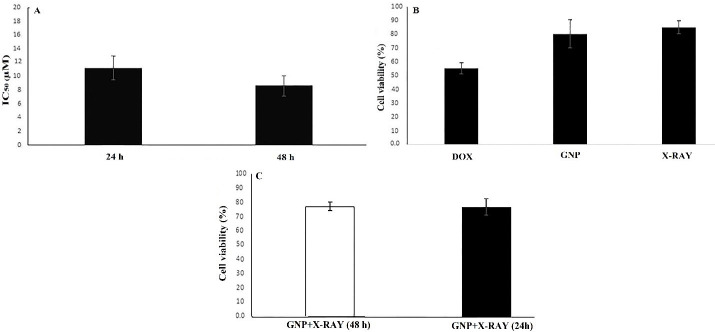
The results of cell viability and IC_50_ values using the WST-1 assay. The IC_50_ values of DOX after 24 and 48 h treatment times (**A**). Cytotoxic effects of single treatments including GNP (at a concentration of 70µM), DOX (at IC_50 _concentration), and 2 Gy of X-ray irradiation after 48 h treatments (**B**). The cytotoxicity of double treatment including GNP + X-Ray after 24 and 48 h treatments (**C**). Data were presented as mean ± SD. GNP; Folic acid conjugated-gold nanoparticles, X-Ray: X-Ray irradiation, DOX; Doxorubicin

To detect the minimally toxic concentration of GNP-F, the cytotoxic effects of GNP-F after 24 and 48 hours treatments were examined. The dose-response curves of GNP-F after 24 hours and 48 hours treatments were plotted (Figure 1). The minimal cytotoxic effect of GNP-F was obtained at a concentration of 70 µM. Therefore, the concentration of 70 μM of GNP-F was selected for double and triple combinations of GNP-F with DOX and X-Ray irradiation. Based on the data of dose-response curves and Compusyn software, a higher IC_50_ value of DOX was obtained after 24-h exposure compared to 48 hours treatment. The IC_50_ values were plotted in Figure 2A.

In order to evaluate the possible cytotoxic effects of X-ray, GNP-F, and DOX in various treatments, we used cell viability assay after 24 and 48 hours treatments. The low concentrations of DOX (0.5 × IC_50_ and 0.25 × IC_50_ for double and triple treatments) were used to observe the efficiency of combinatorial treatments. The results of the WST-1 assay showed that the viability of X-ray treated cells alone was not significantly different compared to the control group after 48 h. Cell viability data were presented in Figure 2.

Further analysis in double combination treatments showed that the cytotoxic effect of the X-ray + GNP-F group was higher than that of related single treatments, but did not reach a significant level after 24 and 48 hours treatments (Figure 2).

On the other hand, X-Ray + DOX (at 0.5 × IC_50_ concentration of DOX) exhibited higher cytotoxicity compared to X-ray irradiation alone after 24 hours. Indeed, There was no significant difference in the viability of CRC cells treated with DOX (0.5 × IC_50_ dose) + GNP compared to IC_50_ concentration of DOX after 24 hours treatment. Double combinations of Dox + GNP-F and X-Ray + DOX showed higher cytotoxicity compared to X-Ray + GNP-F after 24 hours (*P* > 0.05). The cell viability in the double combination group of DOX + GNP-F was lower than that of the X-ray + GNP-F group after 48h treatment

**Figure 3:**
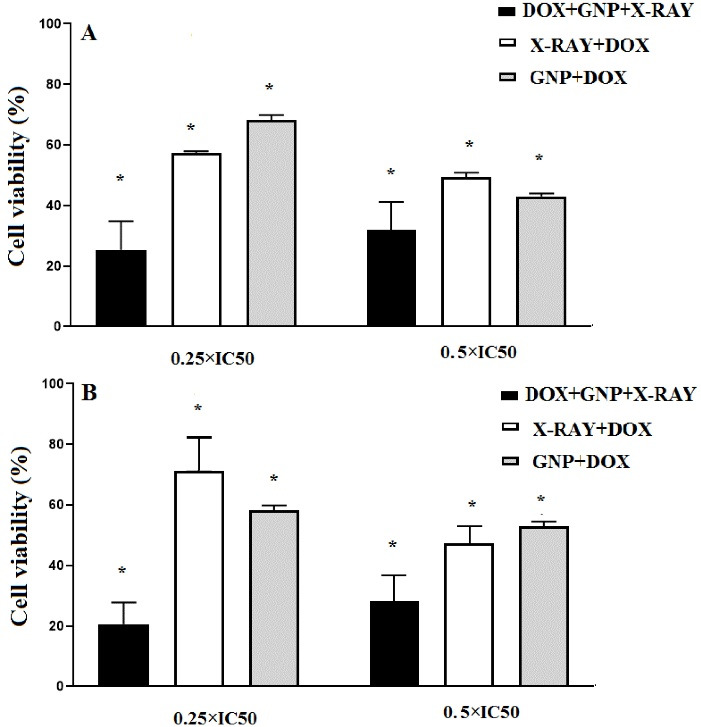
Results of cell viability assay in double and triple combination treatments, which DOX were utilized at concentrations of 0.5 × IC_50_ and 0.25 × IC_50_ after 24 h (A) and 48 h treatments(B). In all combined treatments, 70 µM concentration of GNP-F and 2Gy of X-ray irradiation were used. Data were expressed as mean ± SD. GNP; Folic acid conjugated-gold nano particles, X-Ray: X-ray irradiation, DOX; Doxorubicin. * *P*<0.05 (significant differences compared to the untreated control group)

As presented in Figure 3, double combination treatments that involved DOX at the concentrations of 0.5 × IC_50_ had a higher cytotoxic effect than that of DOX at 0.25 × IC_50_ concentration. Also, in triple combination treatments that DOX was used at 0.25 × IC_50_ concentration, the higher cytotoxicity was observed than that of 0.5 × IC_50_ concentration. The cytotoxic effects of GNPs (70µM) and DOX (0.25 × IC_50_) in combination with X-ray irradiation in HT-29 cells were higher than the related single treatments after 24 hours (*P*<0.05). The results showed that cellular viability in the triple combination group of X-ray + GNP-F + DOX (0.25 × IC_50_) significantly decreased after 48 h compared to all single and double combination treatment groups.

**Figure 4:**
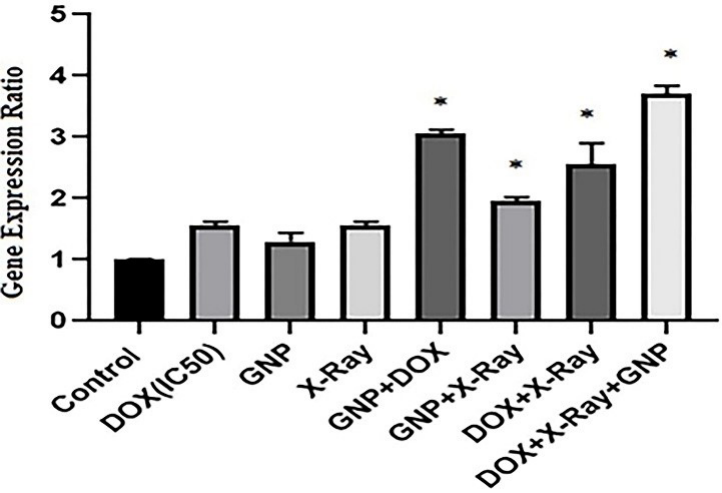
Caspase 3 gene expression ratio in single, double, and triple combinations of X-Ray, GNP-F, and DOX which determined by the real-time PCR method. DOX at the concentrations of IC_50_ (8.6 µM), 0.5 × IC_50_ (4.3 µM), and 0.25 × IC_50_ (2.15 µM) were used in single, double, and triple treatments, respectively (after 48 h). The concentration of 70µM for GNP-F and 2 Gy of x-ray irradiation were used for all combination treatments. GNP-F; Folic acid conjugated-gold nanoparticles, X-Ray: X-ray irradiation, DOX; Doxorubicin. Data were presented as mean fold change ± SD. *P<0.05 (showed a significant difference compared to the untreated control group)

To evaluate the possible molecular mechanisms of cytotoxic effects of GNP-F, X-ray irradiation, and DOX in single and combined treatments, the caspase 3 gene expression ratio as an apoptosis-related gene was evaluated by real-time PCR method after 48 hours treatment. As presented in Figure 4, results of caspase 3 gene expression showed that in all single treatment groups, the caspase 3 mRNA expression increased versus untreated controls, but did not reach the significant level (P > 0.05). In all double and triple combination treatment groups, the caspase 3 mRNA was upregulated compared to uninduced control cells (Figure 4). In addition, in the triple combination treatment group, the caspase 3 gene expression ratio significantly increased compared to single treatments (*P*<0.05).

DOX + X-ray and GNP-F + DOX combination treatment groups had significantly higher caspase 3 mRNA levels than that of single treatments (*P*<0.05). Other double combination treatment did not show a significant increase in caspase 3 mRNA level compared to single treatments (*P* > 0.05).

Likewise, in the triple combination treatment, the caspase 3 gene expression rate increased significantly compared to DOX + X-ray and GNP-F + X-ray double combination treatment groups (*P*<0.05). GNP-F + X-ray combination showed a lower caspase 3 mRNA level compared to the GNP-F + DOX group.

**Figure 5:**
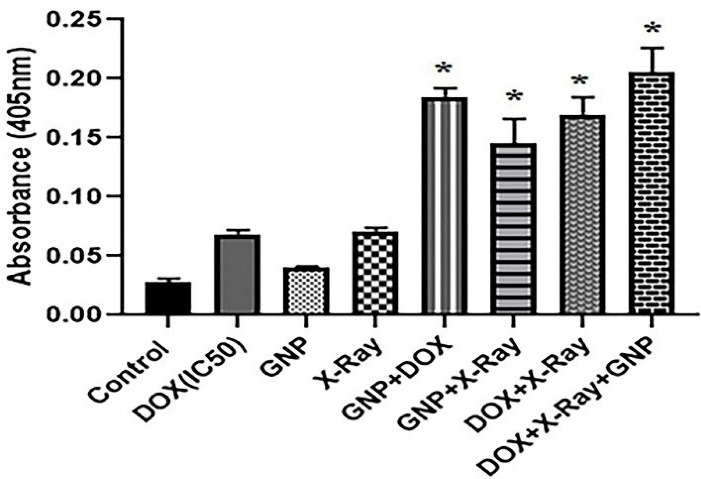
The level of caspase 3 activity in single, double, and triple combinations of X-ray, GNP-F, and DOX. DOX at the concentrations of IC_50_ (8.6 µM), 0.5 × IC_50_ (4.3 µM), and 0.25 × IC_50_ (2.15 µM) were used in single, double, and triple treatments, respectively (after 48 h). Also, the concentration of 70 µM for GNP-F and 2 Gy X-ray irradiation were utilized for all combination treatments. GNP-F; Folic acid conjugated-gold nanoparticles, X-Ray: X-ray irradiation, DOX; Doxorubicin. Data were presented as mean fold change ± SD. * *P*<0.05 (showed a significant difference compared to the untreated control group)

In order to investigate the possible molecular mechanisms involved in the cytotoxicity of GNP-F, DOX, and X-ray irradiation in various treatments, the caspase 3 protein activity as a major marker of apoptosis induction was evaluated (Figure 5). In these treatments, DOX at the concentrations of IC_50_ (8.6 µM), 0.5 × IC_50_ (4.3 µM), and 0.25 × IC_50_ (2.15 µM) were used in single, double, and triple treatments, respectively. Moreover, in all treatments, 2 Gy of X-ray irradiation and concentration of 70µM of GNP-F were used. Based on the results, the caspase 3 activity increased in all double and triple combinatorial treatments in comparison with the control group (P<0.05).

In addition, the caspase 3 activity in double and triple combination groups were higher than single treatment groups (*P* < 0.05). Likewise, in the triple combination treatment, a significantly higher caspase 3 activity was presented compared to the GNP-F + X-ray combination group (*P*<0.05).

**Figure 6:**
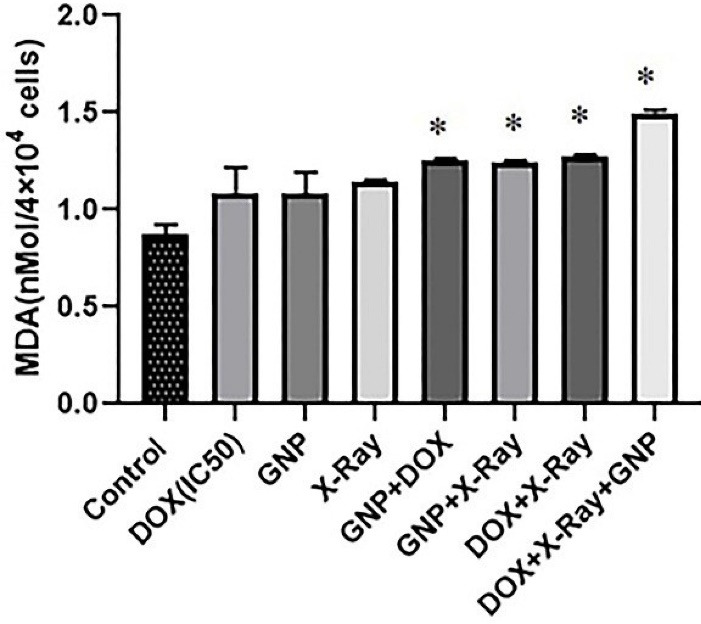
MDA levels in various single, double, and triple combinatorial treatments of DOX, X-Ray, and GNP-F in HT-29 cells after 48 h of treatment. GNP-F; Folic acid conjugated-gold nanoparticles, X-Ray: X-ray irradiation, DOX; Doxorubicin. Data were presented as mean ± SD of independent tests. * *P*<0.05 (showed a significant difference compared to the untreated control group)

As presented in Figure 6, the MDA levels increased insignificantly in single treatments compared to the control group (*P* > 0.05). While double and triple combination treatments increased the MDA levels compared to the untreated control group (*P* < 0.05). There were no significant differences between double combination treatments in comparison with single treatments(*P* > 0.05). There was an increase in the MDA level in the triple combination group compared to single treatments.

According to our results, GNP-F in combination with a low concentration of DOX increased the X-ray-associated cytotoxicity by inducing caspase 3 gene expression and protein activity, which are related to apoptosis induction. In addition, this combination treatment increased the MDA level as a lipid peroxidation marker. Indeed, the combination of GNP-F, DOX, and X-ray irradiation showed noticeable cytotoxic effects compared to untreated controls and single treatments in HT-29 cells. These results indicated that these therapy methods could improve the treatment outcome of CRC cells. Since the cytotoxic effects of the GNP-F + DOX double combination treatment were more than the GNP + X-ray treatment, we next examined triple combinations of X-ray irradiation with GNP-F and a very low concentration of DOX (0.25 × IC_50_). Results showed that the cytotoxicity of the DOX + GNP-F increased significantly when combined with irradiation.

 In a single exposure, X-rays did not induce any significant cytotoxicity in the HT-29 cells compared to the untreated control cells after 48 h of treatment. HT-29 cells treated with the combination of X-rays and GNP-F showed little reduction in cell viability compared to single treatments. In addition, an insignificant increase in caspase 3 mRNA level was presented in these single treatments while cells treated with X-ray + GNP-F + DOX showed significant cell-killing effects and upregulated caspase 3 mRNA as an apoptosis-related gene.

Many studies based on several assays demonstrated that the use of GNPs in the presence of orthovoltage (kV) and mega-voltage (MV) radiation had radiosensitization effects.^13,23-25^

It has been indicated that radiobiological parameters of the survival curve can predict cell radiosensitivity. In this regards, Arab-Bafrani et al, in a similar survey, showed that cell survival in the presence of GNPs was more than 90% and the maximum uptake of GNPs was observed at 60 μM concentration of GNP.^25^ In our survey based on the WST-1 results, we observe minimal cell toxicity against HT-29 cells at the concentration of 70 µM GNP-F.

Various chemotherapeutic drug and nanoparticle conjugate have been stated; nevertheless, few of these have been utilized as a radiosensitizer agent. Indeed, conventional chemotherapeutic drugs often act as radiosensitizers, and maybe just showed the emerging state of the field.^26^

For example, in a study by Starkewolf et al, X-ray irradiated nanoparticle carriers containing DOX conjugated to DNA strands attached to the GNP surface on cancer cells showed the cell cytotoxicity effects.^27^ In another study by Xu et al, gold nanorods conjugated with Arg-Gly-Asp peptides had radiosensitization effects on melanoma A375 cells by decreasing the expression level of Integrin α (v) β₃.^28^

 Based on our results, GNP-F at a concentration of 70 µM in combination with DOX increased the cytotoxic effects of X-ray irradiation. Combination of GNP and DOX increase the radiation effect and cytotoxicity versus control cells.

The important physical mechanisms that describe the radiation interaction with nanoparticles may be related to the Compton and Photoelectric effects. On the other hand, previous studies indicated that there was an increased ROS production in the presence of GNPs. ROS can oxidize the mitochondrial membrane and subsequent cause DNA damage.^29^

GNPs can sensitize cancerous cells to radiotherapy via elevating the ionizing energy deposition in their immediate vicinity. Furthermore, radiation damage is mainly enhanced by ROS, which are generated following water radiolysis. In the current study, radiation-responsive PEGylated GNP were synthesized and could radiosensitize cancer cells by elevating cellular apoptosis after radiation therapy.^30^ Another related study showed that in addition to the immediate photothermal cell damage, elevated concentrations of ROS were generated in irradiated cells.^31^

ROS can damage malignant cells in numerous ways, including DNA damage and modulating gene transcription. The main produced ROS comprises hydrogen peroxide (H2O2,), hydroxyl reactive radicals (-OH), and anion superoxide (O_2_^-^). The degree of damage induced by ROS can be determined based on the amount and type of ROS produced, and the exposure duration to ROS.^32^ After the induction of the high amount of ROS, it can trigger the cell cycle redistribution and cellular apoptosis. The presence of radiosensitizers in the cancer cells could increase ROS generation.^33^ These effects may be related to apoptosis induction, which was observed in this study.

Indeed, balance among apoptosis induction and its inhibition has a critical role in homeostasis of various tissues. Definitely, any dysregulation in apoptosis pathway are relate to pathologic states.^34^ In agreement with our results, Moradi et al showed the radiosensitizing effects of GNP in combination with a chemotherapeutic agent which could increase the apoptosis induction of CRC cells.^35^ Similarly in this study, caspase 3 protein expression was upregulated in CRC cells.^35^

Also in a similar study, when silver nanoparticles (in minimal cytotoxic concentration) combined with a chemotherapeutic agent and 2 Gy of X-ray irradiation could increase the level of H2O2 as an oxidative stress marker and induce apoptosis in the breast cancer cell line.^36^

In the present study for describing one of the possible underlying mechanisms of cytotoxicity of the triple combination treatment, the MDA level was measured. According to the results, the triple combination treatment with significant cytotoxic effects increased the MDA generation level in compared to single treatments. Definitely, MDA is considered a significant product of lipid oxidation. ROS can promote lipid peroxidase, damage the bilayer arrangement of membrane lipids, and increase the permeability of tissues. Indeed lipid peroxidation products, including MDA, can inactivate several cell proteins.^37^ Likewise, in our study, increased MDA level in a triple combination treatment may be related to lipid peroxidation due to increased ROS level.

## Conclusion

In this study, we aimed to evaluate the effects of DOX and GNP-F in combination with radiotherapy in CRC cells. According to our results, GNP-F in combined with X-ray-irradiation and DOX induced significant cytotoxic effects in HT-29 cells. In addition, caspase 3 gene expression ratio, caspase 3 activity, and MDA generation level increased in the triple combination treatment compared to controls. Definitely, a low dose of radiation and GNP-F when combined with DOX at the concentration of 0.25 × IC_50_ induced significant cytotoxic effects. Results of this study might suggest a potential treatment option to increase the efficiency of radiotherapy. Consequently, the results of the present study may be useful in the understanding of the molecular mechanism and interactions between GNP-F and DOX as radiosensitizer agents to enhance the outcome of CRC therapy. However, further studies about combinations of GNP-F, DOX, and radiotherapy should be performed *in vitro* and *in vivo* to confirm their efficiency

## Ethical Issues

 The study protocol was reviewed and approved by the ethical committee of Urmia University of Medical Sciences, (IR.umsu.rec.1395.189).

## Conflict of interest

 The authors declared no conflict of interest.
